# Intervertebral Disc Swelling Demonstrated by 3D and Water Content Magnetic Resonance Analyses after a 3-Day Dry Immersion Simulating Microgravity

**DOI:** 10.3389/fphys.2016.00605

**Published:** 2016-12-05

**Authors:** Loïc Treffel, Karen Mkhitaryan, Stéphane Gellee, Guillemette Gauquelin-Koch, Claude Gharib, Stéphane Blanc, Catherine Millet

**Affiliations:** ^1^Institut Pluridisciplinaire Hubert Curien, Université de Strasbourg, Centre National de la Recherche Scientifique (CNRS)Strasbourg, France; ^2^Siemens Healthinners, Service ApplicationSaint-Denis, France; ^3^CHU Toulouse Rangueil Hospital Imaging ServiceToulouse, France; ^4^French Space Agency, Centre National d'Etudes SpatialesParis, France; ^5^Faculté de Médecine et d'Odontologie, Université Claude Bernard Lyon 1Lyon, France; ^6^Service d'Odontologie, Hospices Civils de LyonLyon, France

**Keywords:** disc swelling, vertebral deconditioning, disc herniation, back pain, dry immersion, microgravity, magnetic resonance imaging/magnetic resonance spectroscopy

## Abstract

**Background:** Vertebral deconditioning is commonly experienced after space flight and simulation studies. Disc herniation is quadrupled after space flight.

**Purpose:** The main hypothesis formulated by the authors is that microgravity results in intervertebral disc (IVD) swelling.

**Study Design:** The aim of the study was to identify the morphological changes of the spine and their clinical consequences after simulated microgravity by 3-day dry immersion (DI). The experimental protocol was performed on 12 male volunteers using magnetic resonance imaging and spectroscopy before and after DI.

**Methods:** All the experiment was financially supported by CNES (Centre national d'études spatiales i.e., French Space Agency).

**Results:** We observed an increase in spine height of 1.5 ± 0.4 cm and a decrease in curvature, particularly for the lumbar region with a decrease of −4 ± 2.5°. We found a significant increase in IVD volume of +8 ± 9% at T_12_-L_1_ and +11 ± 9% at L_5_-S_1_. This phenomenon is likely associated with the increase in disc intervertebral water content (IWC), 17 ± 27%. During the 3 days in DI, 92% of the subjects developed back pain in the lumbar region below the diaphragmatic muscle. This clinical observation may be linked to the morphological changes of the spine.

**Conclusions:** The morphological changes observed and, specifically, the disc swelling caused by increased IWC may contribute to understanding disc herniation after microgravity exposure. Our results confirmed the efficiency of the 3-day DI model to reproduce quickly the effects of microgravity on spine morphology. Our findings raise the question of the subject selection in spatial studies, especially studies about spine morphology and reconditioning programs after space flight. These results may contribute to a better understanding of the mechanisms underlying disc herniation and may serve as the basis to develop countermeasures for astronauts and to prevent IVD herniation and back pain on Earth.

## Introduction

After space flight, astronauts frequently experience a vertebral deconditioning that is characterized by spine lengthening, herniated discs, muscle atrophy, and back pain (Hutchinson et al., 1995; Cao et al., 2005; Belavy et al., 2016). The underlying pathophysiology of intervertebral disc (IVD) herniation in astronauts is, therefore, a research priority for numerous space agencies. Other related research priorities include identification of predisposing factors and development of countermeasures to reduce or prevent this phenomenon after space flight (Belavy et al., 2016).

Intervertebral disc (IVD) herniation appears at recovery from space flight and is more frequent in returning astronauts when compared to the frequency in the general population on Earth, with astronauts experiencing 21 times more IVD herniation in the cervical region and 3 times more herniation in the lumbar region (Belavy et al., 2016). Despite such health issue with the high incidence rate of IVD herniation in astronauts, it is surprisingly that a single study on whole the rachis, particularly on the cervical region during space flight or simulation exists.

The main hypothesis found in the literature on the cause of IVD herniation is IVD swelling. Indeed, studies have demonstrated (Johnstone et al., 1992; Campana, 2004) that the fluid content of the disc, which governs its mechanical response and biological behavior, varies with external load. When the load decreases, liquid is reabsorbed into the IVD to reach a new osmotic equilibrium (Johnstone et al., 1992). Thus far, an overhydrated disc has not been thoroughly evaluated after exposure to microgravity (Adams and Hutton, 1982; Young and Rajulu, 2011; Belavy et al., 2016; Hargens and Vico, 2016).

The aim of the study was to analyze spinal changes during 3 days of simulated microgravity using dry immersion (DI) as the analog (Navasiolava et al., 2011). We hypothesized that (1) a 3-day DI could lead to morphological changes on the spine, which could explain the vertebral deconditioning described by astronauts after space flight; (2) the disc swelling induced by simulated microgravity is explained by an increase in IVD water content; and (3) this phenomenon is likely associated with deep muscle atrophy and could lead to postural balance disturbances, as demonstrated by another team that experienced the same conditions (Treffel et al., 2016).

## Materials and methods

### Subjects

Twelve healthy male subjects between the ages of 26 and 39 (mean ± standard error of the mean as calculated based on data from the day before DI; age: 31.8 ± 4.1 years; height: 178.8 ± 5.6 cm; weight: 74.8 ± 5.6 kg; body mass index: 23.6 ± 1.2 kg/m^2^; aerobic fitness: 38.8 ± 2.9 mL.kg^−1^.min^−1^) participated in this study. No subjects had vertebral transitional anomalies, scoliosis, or any vertebral pathology on study enrollment. Only one subject was excluded from our measurements because of cervical fracture history. All participants were non-smokers, were not taking any medications or drugs, and received a comprehensive clinical assessment before providing their written informed consent. The subjects were selected based on a normal clinical investigation consisting of a detailed medical history, physical examination, an electrocardiogram, general blood screening, and urine analyses. Participants were free from muscular or neurological pathologies.

The study design was established in accordance with the Declaration of Helsinki and was approved by the Comité de Protection des Personnes (CPP Sud-Ouest Outre-Mer I, France) as well as the French Health Authorities under the number ID RCB: 2014-A00904-43. The experimental protocol was organized by the Institute for Space Medicine and Physiology (MEDES-IMPS) in Toulouse, France. All subjects signed an informed written consent prior to the study.

### General protocol

**Figure 1:**
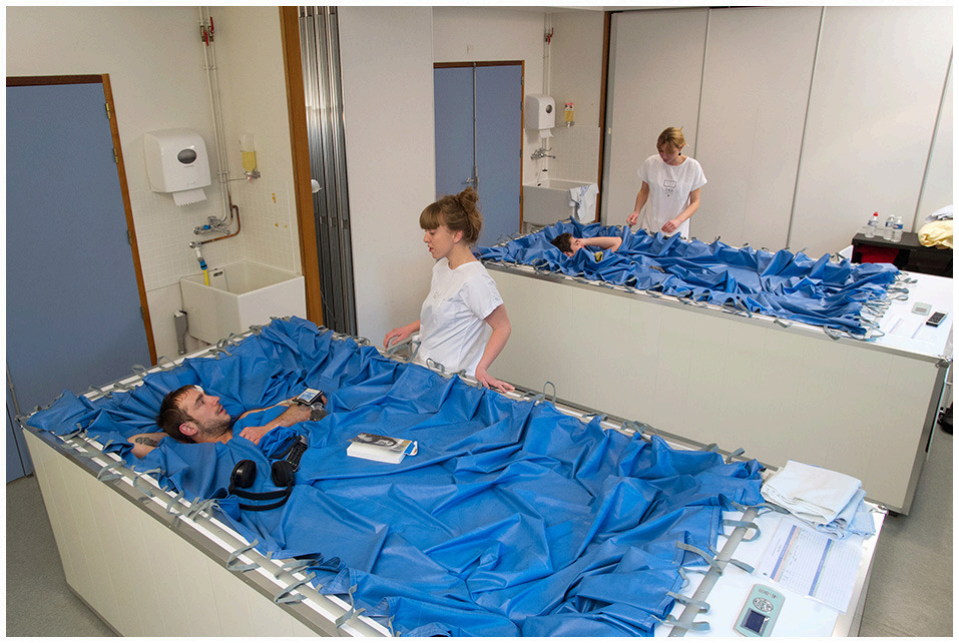
**Dry immersion condition. © CNES/MEDES/E.GRIMAULT, 2015**.

This experiment consisted of a 3-day baseline data collection period (BDC-3 to BDC-1), 3 days of dry immersion (DI 1 to DI 3), and 2 days of recovery (R0 to R+1). In the ambulatory and control periods before and following DI, all subjects remained active and ambulatory. All were asked not to exercise during the 8 days of the experiment. During DI, the subjects remained permanently immersed in a resting and supine position (Figure 1) in a controlled thermo-neutral bath (33 ± 0.5°C), except for a daily 20-min extraction for toilet use and weighing (in the supine position). The study was conducted in a quiet room at a temperature of ~25°C. During this study, eight different research groups performed their protocols on several physiological systems. The different protocols and hygienic procedures involved a total duration of 4 h 45 min out of immersion between DI 1 and R0. During this period the subjects were maintained in a −6° head-down position; the −6° angle was necessary to keep the thoraco-cephalic fluid shift achieved during DI.

During DI, only the head and neck were not entirely immersed. The subjects were supervised by physicians and monitored 24 h per day. Room lighting was on between 7:00 a.m. and 11:00 p.m. Each subject had a daily medical examination, and MEDES personnel took several standardized measurements. Discomfort and psychological assessments were made via questionnaires. Body temperature was taken twice daily with a tympanic thermometer. Heart rate and arterial blood pressure (systolic, mean, and diastolic) were measured every morning by means of an automated sphygmomanometer (Dinamap). The plasma volume was also estimated in the morning (1 h after a light breakfast) in the supine position just before DI (DI 1) and immediately at the end of the DI period, just before standing (R0), using the optimized CO-rebreathing method as described by Caiani et al. (2014).

### Spine protocol

#### Pain assessments

A visual analog scale (VAS) was used to identify the intensity of back pain experienced by the subjects in the cervical, thoracic, and lumbar regions. The same medical doctor performed all the measurements of pain 2 days before the start of the DI period (BDC-2) and on the first day of the recovery period (R0). Additionally, a discomfort score was determined every morning and evening during the DI experiment. Forward flexion of the spine was determined by measuring the distance between the ground and the patient's fingers (hand-to-ground distance).

#### Magnetic resonance imaging

Magnetic resonance imaging (MRI) was performed on the patients in the horizontal position using a Siemens Magnetom Avanto Syngo MR B17, TR 1200 ms, TE 119 ms, slice 1 mm. The subjects were transferred 10–15 feet from the DI room to the MRI facility in the head-down bed-rest position in a −6° decline. The MRI analysis was performed using the OsiriX MD v.7.0.1 64-bit software.

**Figure 2:**
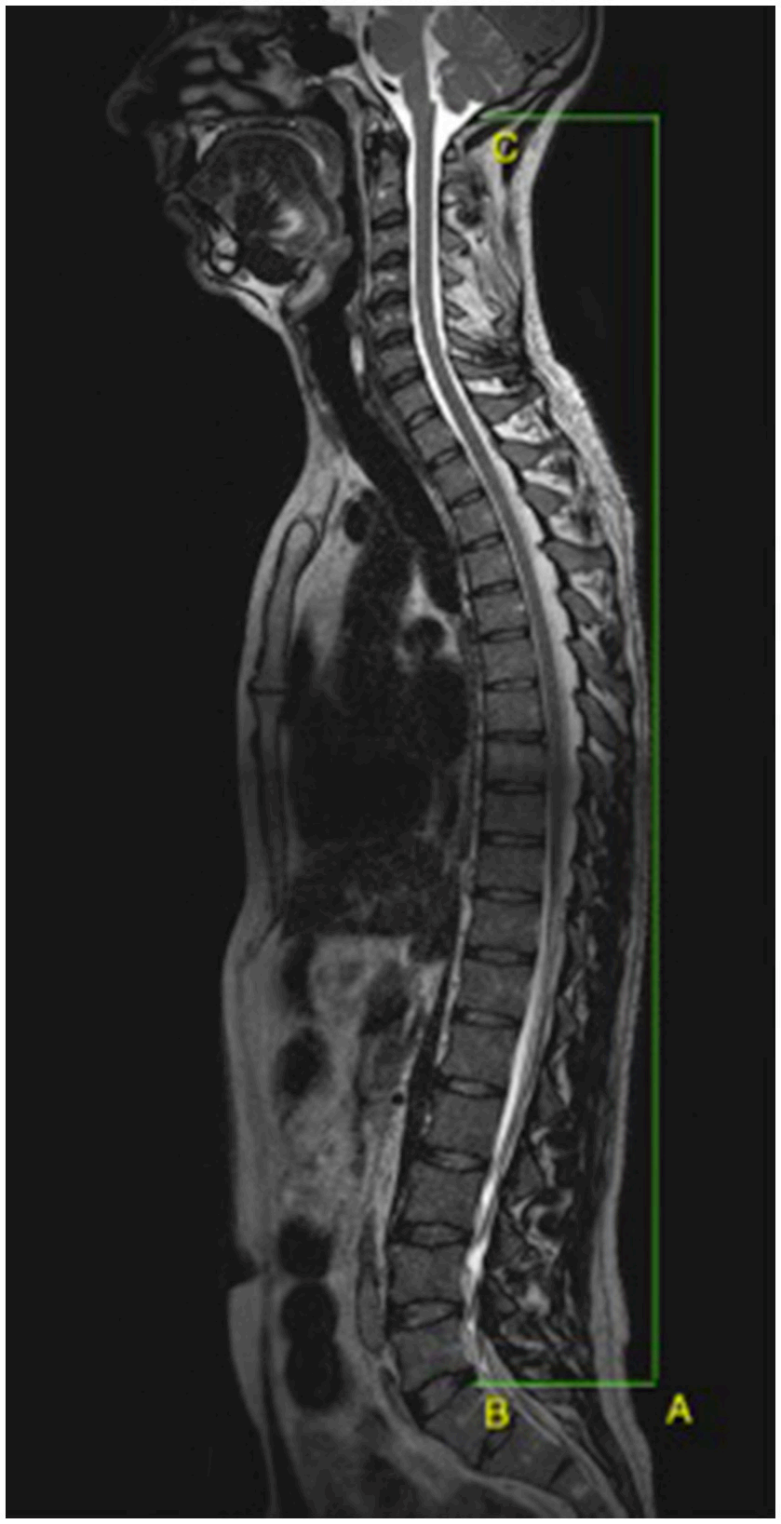
**Spine height measurement (A)** between the foramen magnum **(C)** and the superior and posterior endplate of the first sacral vertebra **(B)**.

*Height of spine* (Figure 2) was measured on sagittal T2 3D MR images (Repetition time: 1200 ms, Echo time: 119 ms, FoV: 400 mm, slice 1.00 mm, flip angle: 125°, field of view: 587 hertz/pixels) between the foramen magnum of the occipital bone (C_0_) and the posterior-superior endplate of the first sacral vertebra (S_1_). We used the median sagittal plane, crosschecked against the horizontal plane at the same level of spine (superior endplate of C_3_), to perform the measurements with the same landmark before and after DI.

*Curvature* was measured in the median sagittal plane in lumbar (L_1_-S_1_) using the method described by Andersson et al. (1979); the method was also applied for the thoracic level (T_1_-T_12_). In the cervical region, the angle between C_0_, anterior-superior angle of C_4_ endplate, and upper endplate of T_1_ was measured using MRI T2 3D sagittal and axial sequences centered in the median sagittal plane using the middle of the medullar canal.

*Intervertebral disc volume* was measured using the horizontal crosschecking sagittal and axial sequences on T1 vibe Dixon axial in phase opposition, where the IVD appears in hyper-signal (**Figure 8**). On each slice, the disc signal was selected as the region of interest (ROI). The volume was calculated using all the ROIs with OsiriX MD software in a three-dimensional reconstruction.

We used a 3D MRI analysis of IVD compared to classical measurements (Hurxthal, 1968; Belavý et al., 2013). As Belavy described in 2013 (Belavý et al., 2013) on analysis of the cervical region, the following method was used to reduce measurement error due to the small size of the cervical discs (C2/3 and C7/T1): (1) each parameter was measured twice in all images, and (2) if the average value for a given parameter from all images at a given IVD varied by more than 3%, then the operator repeated the measurements until agreement was within the 3% threshold.

#### Magnetic resonance spectroscopy

**Figure 3:**
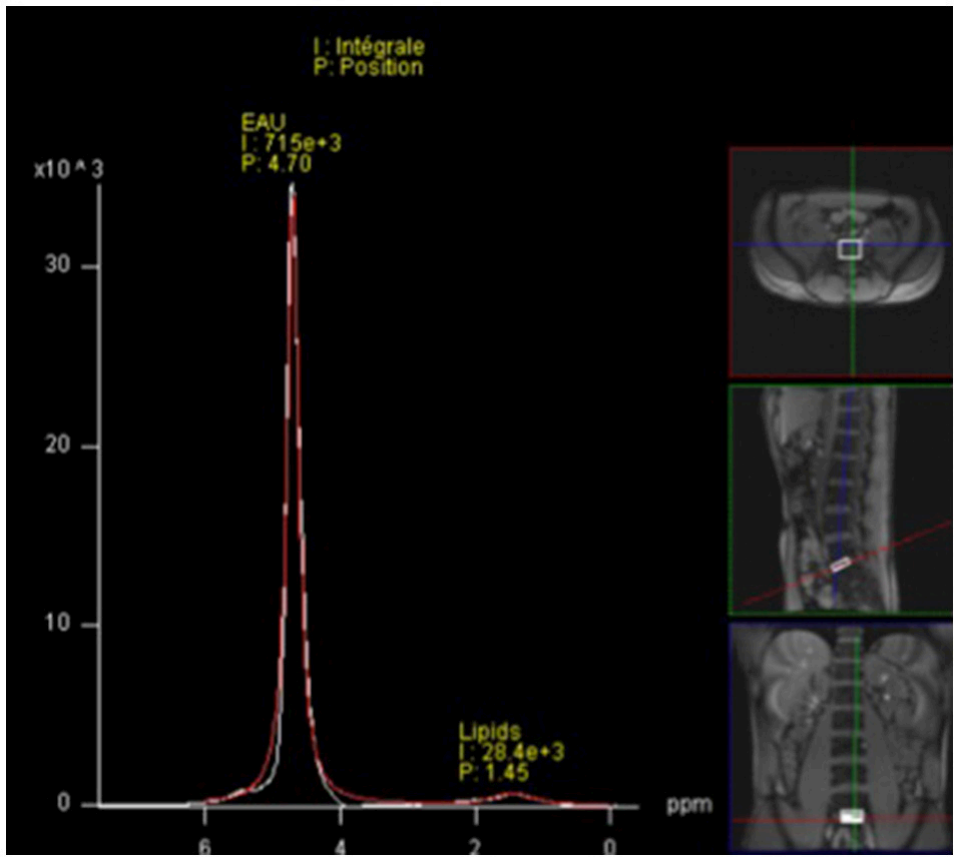
**Intervertebral disc water spectrum analysis: example with the voxel of interest in L_**5**_-S_**1**_, position (P) of water spectrum P_**w**_ = 4.7; integral (I) represents the water content calculated under the peak spectrum**.

We used magnetic resonance spectroscopy to measure the intervertebral water content (IWC) inside the IVDs at four levels, C_2_-C_3_, C_7_-T_1_, T_12_-L_1_, and L_5_-S_1_. These levels were chosen to minimize the time spent by subjects in MRI examination. Mechanically, we selected these regions because they represent the areas that undergo physiological change in vertebral curvatures. Imaging parameters were Echo time: 30 ms, repetition time: 1500 ms, voxel of interest (VOI): 18 × 20 × 5 mm for C_2_-C_3_ and C_7_-T_1_, and VOI: 25 × 30 × 7 mm for T_12_-L_1_ and L_5_-S_1_. VOI were selected crosschecking the three planes of 3D analyses in order to measure the water content inside the disc. Then, the IWC was evaluated by a spectrum analysis using the physiological position of water spectrum (P) Pw = 4.7 ppm as the reference (Figure 3).

### Statistical analysis

The present study was designed to examine the effects of 3 days of DI on the spine. Analyses were performed using the StatPlus software build 6.0.3/Core v5.9.92. The effects of DI on spinal parameters were evaluated by repeated measures. The paired *t*-test was used to compare the same group before and after DI. A *p* < 0.05 was considered statistically significant.

## Results

### Pain

**Figure 4:**
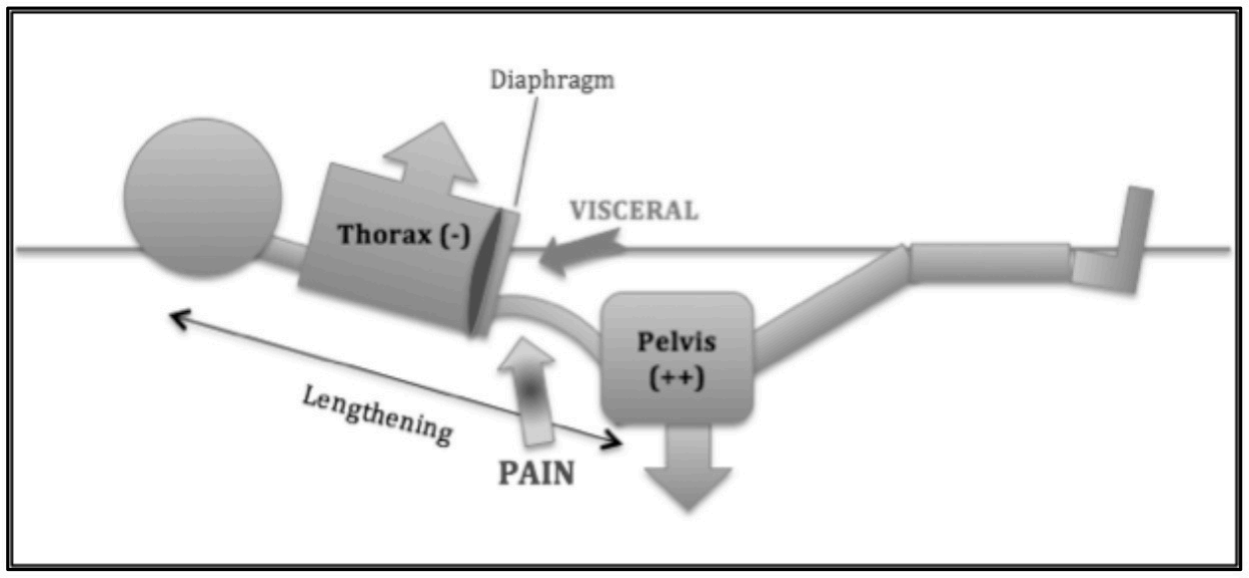
**Position during dry immersion (DI) showing the discomfort or painful sensation**. The most significant discomfort was located in the thoracolumbar region. The pelvis is heavy, so it had a tendency to sink; and the thorax is full of air, so it rose while in DI. Moreover, the viscera draws toward the diaphragm muscle. All these phenomena concentrated constraints in the thoracolumbar region.

**Figure 5:**
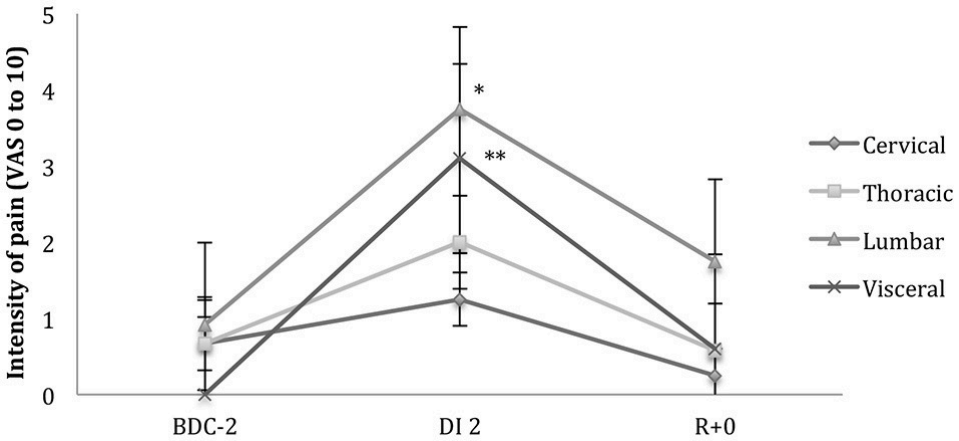
**Pain intensity per region using the visual analog scale (VAS, 0 to 10 where 10 is the worst pain) before (BDC-2), during dry immersion (DI 2), and at recovery (R+0)**. Paired *t*-test (BDC vs. DI), *p*-value significant (^*^p = 0.019; ^**^p = 0.006).

During the study, 92% of subjects developed back pain; and 90% of those had low back pain (Figures 4, 5). Subjects developed back pain during confinement, with the highest intensity of 3.75 ± 2.4/10 in the lumbar region (*p* = 0.012) at DI 2. This intensity decreased significantly at R+0 from 3.75 to 1.75/10 ± 2.2 (*p* = 0.0022) in the lumbar region. Pain intensity decreased at R+0 from 2/10 to 0.6/10 (*p* = 0.06) in the thoracic part of spine. A visceral pain was described, 3.1 ± 2.4/10, during DI for 11 of 12 subjects. The pain decreased significantly, with a mean of 0.6 ± 1.2/10 (*p* = 0.001), at recovery on loading.

### Discomfort

A significant global discomfort was observed in 11 of 12 subjects with a mean intensity of 40 ± 23/100 over the 3 days during DI and R+0 (Table 1). A specific position was involved in the bath (Figure 4), which may have concentrated the constraints on the thoracolumbar region.

**Table 1:** **Clinical observations (Hand-to-ground distance (HTGD), discomfort by visual analog scale (VAS), and spine height by MRI)**.

**Subjects**	**HTGD (cm) (R+0 − BDC-4)**	**Discomfort scale mean during DI**,	**Spine height (cm) (values after – before) (VAS 0–100)**
A	+16	33	+1
B	+10	59	+1.5
C	+21	56	+1.4
D	+14	63	+1.1
E	+15	47	+1.2
F	+3	41	+1.7
G	+4	63	+1.5
H	+11.5	67	+2
I	+12	6	+1.8
J	+15	0	+1
K	+15	27	+2.4
L	+8	21	+1.4
Mean	+12 ± 5 cm (*p* < 0.001)	40 ± 23/100	+1.5 ± 0.4 cm (*p* < 0.001)

*Changes after dry immersion (DI) at recovery (R+0) compared to baseline (BDC-4). Paired t-test in pre vs. post comparison. Pearson correlation HTGD vs. Height: r = 0.21 (p ≤ 0.05)*.

### Hand-to-ground distance

Spine motion in flexion decreased for all the subjects after DI with a significant increase in hand-to-ground distance of +12 ± 5 cm (pre vs. post, *p* < 0.001) (Table 1).

### Plasma volume, plasma Na^+^, estimated plasma osmolarity

The plasma volume decreased significantly by 17%, from 3727.3 ± 109.9 mL at DI 1 to 3095.6 ± 86.7 mL at recovery (*p* < 0.0001). Plasma Na^+^ increased (*p* < 0.001) from BDC-1 (135.9 ± 5.3 mmol/L) to reach a peak at DI 2 (143.9 ± 3.9 mmol/L). Estimated plasma osmolarity increased significantly (*p* < 0.001) at DI 2 (297 ± 7.9 mOsm/L) compared to BDC-1 (280 ± 11.1 mOsm/L).

### Imaging analysis

**Figure 6:**
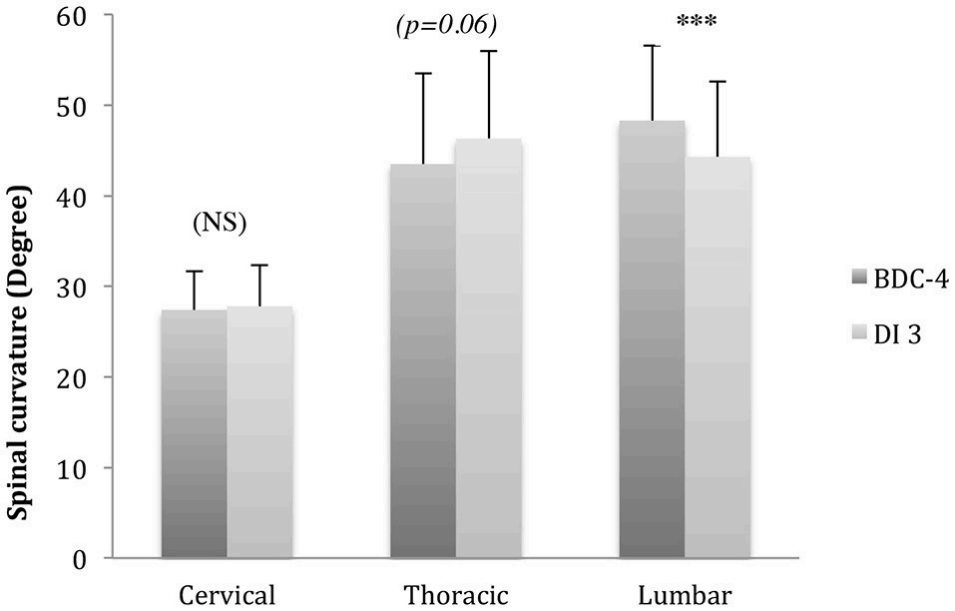
**Spinal curvature (in degree °) changes compared to baseline [before (BDC-4) and after dry immersion (DI3)]**. Lumbar curvature decreased after DI for −8 ± 6% (^***^p < 0.001) (Comparison Pre vs. Post DI, *T*-Paired Test).

**Figure 7:**
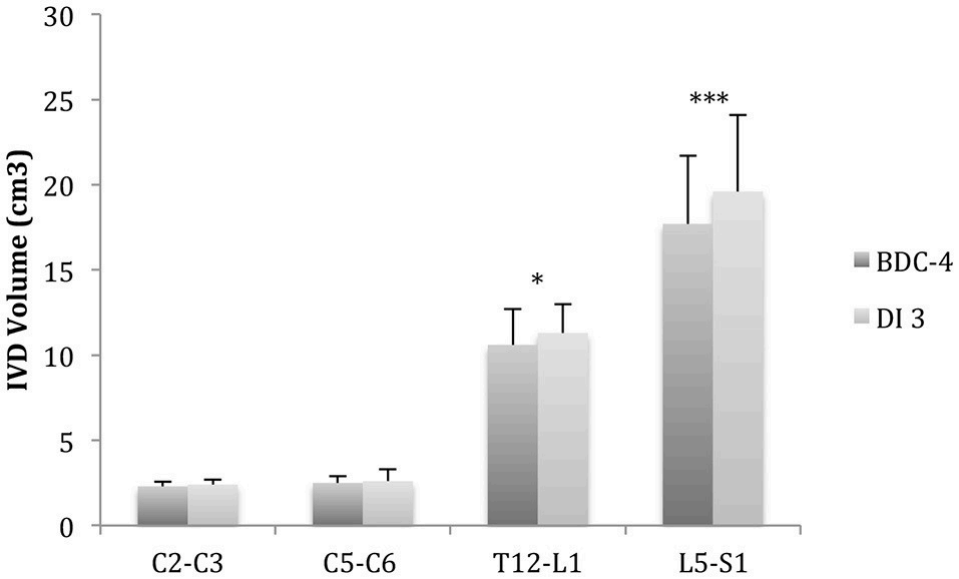
**MRI 3D analysis of intervertebral disc volume (IVD)**. IVD volume increased after dry immersion (DI) in L5-S1 (+11 ± 5%^***^) and in T12-L1 (+8 ± 9%^*^). Comparison pre (BDC-4) vs. post DI (DI3) (Mean ± *SD, T*-Paired test, ^*^p < 0.05; ^***^p < 0.001).

**Figure 8:**
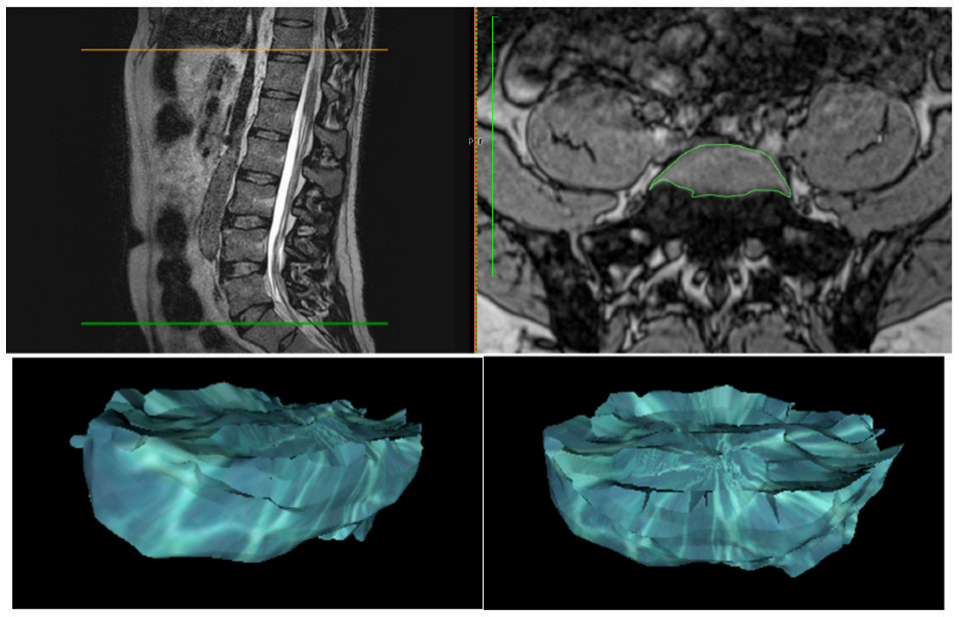
**Intervertebral disc volume 3D reconstruction after regions of interest selected on hyper-signal disc on OsiriX software crosschecking horizontal and axial planes (T1 vibe Dixon opposition phase)**.

Spine height increased significantly, over 1.5 ± 0.4 cm (*p* < 0.001), for all the subjects after 3 days in DI (Table 1). Lumbar curvature decreased significantly with −4 ± 2.5° (*p* < 0.001), resulting in a loss of lordosis (Figure 6). Intervertebral disc volume (IVD) (Figures 7, 8) increased significantly, over 9.5 ± 7% (*p* < 0.001), in the lumbar region. These results indicate a significant lengthening and a straightening of spine after a 3-day DI. We found no significant results in cervical spine pre- vs. post-DI analysis. Spectroscopy analysis showed a significant increase in IWC of over 17% on average after DI (Table 2), except for the C_2_-C_3_ IVD, which showed no significant change following DI.

**Table 2:** **Intervertebral water content calculated by spectroscopy (Water spectrum analysis)**.

	**C_2_-C_3_**	**C_7_-T_1_**	**T_12_-L_1_**	**L_5_-S_1_**	**Average**
Difference between BDC-4 and DI3 (%)	+5	+10	+29	+23	+17
Median position of water spectrum (P_w_)	4.58 ± 0.11	4.70 ± 0.01	4.72 ± 0.07	4.69 ± 0.04	4.67 ± 0.23
Significance (*p* ≤ 0.05)	NS	*p* < 0.05	*p* < 0.01	*p* < 0.01	*p* < 0.05

*Comparison before (BDC-4) and after dry immersion (DI3). Physiological position of water spectrum (P_w_) is P_w_ = 4.7*.

## Discussion

This is the first study to examine the impact on the entire spine of simulated microgravity. These results provide a basis to understand the mechanisms underlying IVD herniation after space flight.

Our findings provide evidence to support the disc-swelling hypothesis formulated previously by researchers (Belavy et al., 2016) during actual microgravity, particularly in the lumbar region. To the best of our knowledge, this is the first 3D-analysis of IVD volume (IVD) in a DI study. Unloading could decrease the intradiscal pressure, which decreases the intradiscal hydrostatic pressure, possibly resulting in a transfer of water into the IVD. Increased water content within the IVD would disturb the osmotic and oncotic equilibrium (Adams and Hutton, 1982; Hargens and Vico, 2016), but we cannot yet confirm this hypothesis. Further investigation is needed to evaluate the simultaneous glycosaminoglycan content (Schmidt et al., 2016; Schleich et al., 2016a,b). Recent studies favor a poro-elastic behavior model of the IVD explaining in and out fluid flow in relation to loading (Vergroesen et al., 2016). In our study, plasma volume and osmolarity variations suggest out-flow of water during loading (Coupé et al., 2013). Therefore, our IVD volume results would validate the mechanical hypothesis that a decrease in hydrostatic pressure leads to a fluid transfer into the IVD.

The overhydrated IVD is a known risk factor for herniated disc (Adams et al., 1987). The disc is then more vulnerable to the herniation mechanism. Flexion bending, associated with compression, is considered a greater risk factor for disc herniation (Wade et al., 2014).

Our results on spine morphology are globally the same as compared to bed-rest studies (Hutchinson et al., 1995; Cao et al., 2005; Pool-Goudzwaard et al., 2015; Belavy et al., 2016), but we were able to obtain our results faster. For example, in Belavy et al. (2016), IVD volume grew by 7% after 60 days in head-down bed rest compared to 9.5% for lumbar discs in our 3-day DI study. Similarly, Cao et al. (2005) found a −3.3° decrease in lumbar curvature following 28 days of head-down bed rest compared to −4° decrease in our 3-day DI study. Comparing our results to those obtained in bed-rest studies validates DI (Navasiolava et al., 2011) as a fast and accurate model to simulate the effects of microgravity on the spine as well as on plasma volume (−17% pre vs. post). DI appears to be an accurate model to simulate increased IVD volume caused by microgravity except in the cervical region, which is not full submerged in the DI bath. DI also creates specific constraints on the body because of the specific position (Figure 4).

In our opinion, pain and disc herniation could result during recovery on loading following exposure to microgravity because of instability. Morphological changes of the spine, including the overhydrated disc, and muscle atrophy could increase markedly the probability of developing a disc herniation and back pain. The constraints elicited on the “unstable spine” at recovery could explain IVD herniation. This vertebral deconditioning observed after microgravity could lead to postural disturbances (Huang et al., 2016; Treffel et al., 2016). Further research on these relationships is needed.

We would like to formulate a functional hypothesis for the vertebral region to identify the predisposing factors for pain and disc herniation and to propose a countermeasure.

### The lumbar region

Recovery from microgravity results in compressive constraints, which lead to an increase in IVD pressure (Campana, 2004) and a zygapophyseal articular conflict, which are risk factors for disc herniation (Wade et al., 2014; Ghasemi et al., 2016).

As a solution, we propose the following:

- Erector spinae muscles, such as the multifidus, have to be strengthened, with parallel strengthening of the transverse abdominal muscle (Ramos et al., 2016). We could add strengthening of perineal muscles to obtain complete control of abdominal pressure, thereby maintaining good control of the lumbar curvature (Sapsford et al., 2013; Vleeming et al., 2014; Arshad et al., 2016; Ehsani et al., 2016; Giacomini et al., 2016).
- The muscles of the pelvis and legs could compensate for the lumbar instability in the upright position. Moreover, there is direct mechanical information, which is the ground reaction force. All the sensorimotor information would have to be conserved as countermeasures in order to stimulate the mechanical input necessary for upright posture on Earth (Egawa et al., 2000; Reschke et al., 2009; Wood et al., 2015).

### The cervical region

The DI model appears to be inaccurate in simulating microgravity effects on the cervical spine. This could be explained by the emerged position of head during DI. This raises the question of cervical disc herniation in simulation studies and what effect the use of a pillow and the freedom of movement have on the cervical region. Moreover, no countermeasures have been developed for the cervical region. We note that all the measures and devices used as physical countermeasures in space are well-described and designed for the lower limbs and thoracic and lumbar regions. But, to our knowledge, no compression or resistive efforts have been developed for the cervical region.

More generally, we can consider that recovery to Earth's gravity corresponds with hypermobility of the cervical column, which could elicit different mechanical constraints. Moreover, cervical vertebrae are dedicated to mobility, to move the head to provide the individual with a good view of the surrounding environment.

We can add a biomechanical aspect to countermeasures, knowing that the center of gravity for the head is anterior to the spine. Thus, the cervical region tends to flex, which may favor posterior disc herniation. Recovery on Earth requires additional muscular effort to support the head.

Prior research demonstrated an increase in cervical disc herniation in astronauts of 21 times more than in the general population on Earth (Belavy et al., 2016), which could be explained by the impact of gravity on return as a shock wave that extends to the cervical region. Indeed, when the astronaut is running after a space flight, each ground impact has a repercussion in the cervical region, which is not designed to resist a compression force. This raises the question of the post flight reconditioning program (Loerch, 2015; Loehr et al., 2015) and inflight cervical countermeasures to minimize or prevent injury to the cervical region.

## Conclusion

Short-term DI led to significant morphological changes on the spine. This model appears to be very accurate for vertebral deconditioning simulation.

This experiment provided strong data to validate the disc-swelling hypothesis formulated by Belavy et al. (2016), which could explain the increased incidence of IVD herniation described after space flight. The cervical region has to be studied more precisely in the future to prevent this impairment.

Further research into additional hypotheses is needed to understand the mechanisms underlying cervical disc herniation in astronauts. In our DI experiment, we found significant balance-control disturbances (Treffel et al., 2016). This postural deconditioning observed after microgravity exposure could be linked to vertebral deconditioning exposed here. These two phenomena seem to be counteracted by conservation of deep postural muscles like the soleus or multifidus. However, specific exercises focusing on the cervical region are lacking. More attention should be focused on this problem at recovery for astronauts and to deduce more information about IVD herniation on Earth.

## Author contributions

Conception and research design: LT, GGK, CG, SB, CM. Experimental work and data analysis: LT, KM, SG, CG, CM. Drafting, editing, revision and approval of final version of manuscript: LT, GGK, CG, SB, CM.

### Conflict of interest statement

The authors declare that the research was conducted in the absence of any commercial or financial relationships that could be construed as a potential conflict of interest.

## Abbreviations

BDC: Base Data Collection
DI: Dry Immersion
IVD: Intervertebral Disc
IWC: Intervertebral Water Content
MRI: Magnetic Resonance Imaging
R: Recovery.
